# Identification and fine-mapping of a major QTL (*PH1.1*) conferring plant height in broomcorn millet (*Panicum miliaceum*)

**DOI:** 10.3389/fpls.2022.1010057

**Published:** 2022-10-11

**Authors:** Tianpeng Liu, Xueying Liu, Jihong He, Kongjun Dong, Wanxiang Pan, Lei Zhang, Ruiyu Ren, Zhengsheng Zhang, Tianyu Yang

**Affiliations:** ^1^Crop Research Institute, Gansu Academy of Agricultural Sciences, Lanzhou, China; ^2^College of Agronomy and Biotechnology, Southwest University, Chongqing, China; ^3^College of Life Science and Technology, Gansu Agricultural University, Lanzhou, China

**Keywords:** broomcorn millet, plant height, BSA-seq, fine-mapping, *AP2*

## Abstract

The plant height of broomcorn millet (*Panicum miliaceum*) is a significant agronomic trait that is closely related to its plant architecture, lodging resistance, and final yield. However, the genes underlying the regulation of plant height in broomcorn millet are rarely reported. Here, an F_2_ population derived from a cross between a normal variety, “Longmi12,” and a dwarf mutant, “Zhang778,” was constructed. Genetic analysis for the F_2_ and F_2:3_ populations revealed that the plant height was controlled by more than one locus. A major quantitative trait locus (QTL), *PH1.1*, was preliminarily identified in chromosome 1 using bulked segregant analysis sequencing (BSA-seq). *PH1.1* was fine-mapped to a 109-kb genomic region with 15 genes using a high-density map. Among them, *longmi011482* and *longmi011489*, containing nonsynonymous variations in their coding regions, and *longmi011496*, covering multiple insertion/deletion sequences in the promoter regions, may be possible candidate genes for *PH1.1*. Three diagnostic markers closely linked to *PH1.1* were developed to validate the *PH1.1* region in broomcorn millet germplasm. These findings laid the foundation for further understanding of the molecular mechanism of plant height regulation in broomcorn millet and are also beneficial to the breeding program for developing new varieties with optimal height.

## Introduction

Broomcorn millet (*Panicum miliaceum*), an allotetraploid species (2*n* = 4*x* = 36) with two subgenomes originating from *Panicum capillare* and *Panicum repens* (Hunt et al., 2014), has been reported as probably one of the earliest domesticated crops around the world (Lu et al., 2009; Li et al., 2021). Phytolith evidence suggested that the domestication of broomcorn millet in northern China could be dated to approx. 10,300 years before the present (cal yr BP) (Yang et al., 2012). Due to its desirable characteristics including a short growing season, high water use efficiency, and drought tolerance, broomcorn millet has always been an important crop in semiarid regions. The grains of broomcorn millet are rich in minerals, essential amino acids, vitamins, and fatty acid. Especially, the protein content in broomcorn millet is higher than that in rice, maize, wheat, and most other cereals (Das et al., 2019). In addition, broomcorn millet is gluten-free and has a low glycemic index, which makes it an ideal nutritious food for a healthy diet (Das et al., 2019; Zou et al., 2019). However, the yield and the annual production of broomcorn millet are quite lower than those of other cereals. Therefore, the breeding of broomcorn millet with desirable agronomical traits and high production will be of great significance.

Plant height, determined by the length of the internode and the number of nodes, is a vital agronomical trait of crops and an important factor influencing crop yield. Cereal plants with a high culm are usually susceptible to lodging, thus decreasing final yield. For the breeding programs of main cereal crops such as rice and wheat, the dwarf trait has been exploited in the first “green revolution” and led to a rapidly increasing production. Genes in hormone pathways, such as *sd1*, *d35*, and *ddf1* involved in the gibberellin (GA) signaling pathway (Sasaki et al., 2002; Itoh et al., 2004; Magome et al., 2004), *brd1* involved in the brassinosteroid signaling pathway (Makarevitch et al., 2012), and *tdd1* involved in the auxin signaling pathway (Sazuka et al., 2009), have been reported to play roles in the morphogenesis of plant height. However, most of these genes have been identified from rice, wheat, and maize, whereas no candidate gene has been reported in broomcorn millet.

The rapid development of molecular biology and sequencing technologies provided various tools for modern crop breeding. Map-based cloning of genes involving significant agronomical traits and marker-assisted selection (MAS) have been widely applied in crops (Chen et al., 2021; Koppolu et al., 2022). Bulked segregant analysis with whole-genome resequencing (BAS-seq) is an effective approach for the identification of candidate genes and has been successfully utilized (Lee et al., 2020; Wu et al., 2022; Zhang et al., 2022). However, genetic and genomic studies of broomcorn millet were more hysteretic than those of other crops. The release of the assembled genome of broomcorn millet in 2019 made it possible to make full use of modern technologies, including BAS-seq and molecular marker development (Shi et al., 2019; Zou et al., 2019).

In the present study, the broomcorn millet dwarf mutant Zhang778 was crossed with the regular variety Longmi12 to explore the candidate quantitative trait loci (QTLs) and genes responsible for plant height trait. One major QTL, *PH1.1*, was identified and fine-mapped into a 109-kb interval using BSA-seq and linkage mapping analysis. Fifteen genes were located in the candidate region, and three candidate genes were the most likely candidate genes for *PH1.1*. Three markers linked with *PH1.1* were identified through association analysis, which validated the mapping results and could be utilized as diagnostic markers of plant height for broomcorn millet breeding. Our results laid a foundation for understanding the molecular mechanism of plant height morphogenesis and contribute to breeding varieties of broomcorn millet with optimal plant architecture.

## Materials and methods

### Plant materials and phenotypic analysis

Longmi12 and Zhang778 were chosen as the parental lines to establish an F_2_ population in order to analyze the plant height trait of broomcorn millet. Longmi12 is a high-yield variety bred by the Institute of Crop Research in Gansu Academy of Agricultural Sciences, China. The height of mature Longmi12 plants is about 160–180 cm. Zhang778, bred by Zhangjiakou Academy of Agricultural Sciences, China, is a dwarf line created through ethyl methanesulfonate (EMS) mutagenesis, with a final height of about 60–70 cm. Longmi12 was crossed with Zhang778 in Huining in 2018, the F_1_ plants were planted also in Huining in 2019, and the F_2_ population with 939 individuals was planted in the summer of 2020 in Huining, Gansu Province. The progenies of each F_2_ individual were planted in a line to develop the F_2:3_ population in the summer of 2021 in Huining, Gansu Province.

The plant height of broomcorn millet was measured using a ruler with an accuracy of 0.1 cm. For each F_2:3_ line, the average height of all plants per line was taken as a representative value. Moreover, the internode length of adjacent nodes and the panicle length in Longmi12 and Zhang778 were measured.

The R package “SEA V2.0” (https://cran.r-project.org/web/packages/SEA/index.html) was utilized to analyze the potential inheritance models of plant height in the F_2_ population. The optimal inheritance model was selected following the instructions in SEA v2.0.

To verify the accuracy of QTL *PH1.1*, a total of 512 broomcorn millet accessions collected from all over the world were used to carry out an association analysis (**Supplementary Table S1**). These accessions were planted and measured in Huining in 2020 and 2021.

### BSA-Seq and resequencing analysis

The DNAs of Longmi12, Zhang778, and the F_2_ individuals were extracted from young leaves using a modified cetyltrimethyl ammonium bromide (CTAB) method (Zhang et al., 2005). Fifty F_2_ individuals with height over 160 cm were collected for the “high bulk,” (HB), while 50 individuals shorter than 100 cm were collected for the “dwarf bulk” (DB). The DNA libraries of the parents and the two bulks were sequenced on an Illumina HiSeq 2000 platform. Clean reads were aligned to the reference genome assembled by Zou et al. (2019) using BWA software with default parameters (Li and Durbin, 2009). Single nucleotide polymorphisms (SNPs) and insertions–deletions (Indels) were identified using GATK software. The ΔSNP and ΔIndel index values were calculated to obtain the preliminary intervals associated with the plant height of broomcorn millet.

### Marker development, linkage map construction, and fine-mapping

To narrow down the preliminary region obtained from the BSA-seq results, Indels in the candidate region were selected according to the comparative genomic resequencing data between Zhang778 and Longmi12. Indel primers were developed based on the flanking sequence of the target Indels. These primers were first used to screen the parents in order to confirm the polymorphism and then to detect the genotype of the F_2_ individuals.

The software JoinMap 4.0 (Van Ooijen, 2006) was applied to construct the genetic linkage map, while the Kosambi map function was utilized to convert the recombination values into genetic distances (in centimorgan). Fine-mapping analysis of the QTL and the evaluation of its effect were carried out using MapQTL 6.0 (Van Ooijen, 2009) in the F_2_ and F_2:3_ populations independently. For the input trait values, the plant height of each F_2_ individual and the average value of each F_2:3_ line were used. MapChart 2.2 (Voorrips, 2002) was applied to represent the genetic map and QTL information graphically.

### Prediction of the candidate genes

The genome information and the functional annotation of the genes in the candidate region were obtained from the reference genome of broomcorn millet (Zou et al., 2019). The genome resequencing data of Zhang778 and Longmi12 were used to compare the sequence variance between the two parents. Genes with ΔSNP/ΔIndel index values equal to 1 or −1 and are harboring sequence variance in the coding regions or potential promoter regions were predicted to be candidate genes.

### Regional association analysis

Association analysis of the candidate region was conducted using a total of 512 broomcorn millet accessions. DNA extraction was performed for all accessions using the CTAB method modified by Zhang et al. (2005). The Indel markers in the candidate region were utilized to obtain the corresponding genotype of these accessions. According to the method used by Liu et al. (2017), the genotypes of each Indel in all accessions were obtained with PCR amplification and polyacrylamide gels. Thereafter, the accessions were separated into different haplotypes for each locus, and comparisons of the plant height between haplotypes were conducted using the Student’s *t*-test. A significance threshold (*p* < 0.05) was used to determine the significant associations between haplotypes and plant height.

## Results

### Phenotypic characterization and inheritance of plant height

**Figure 1:**
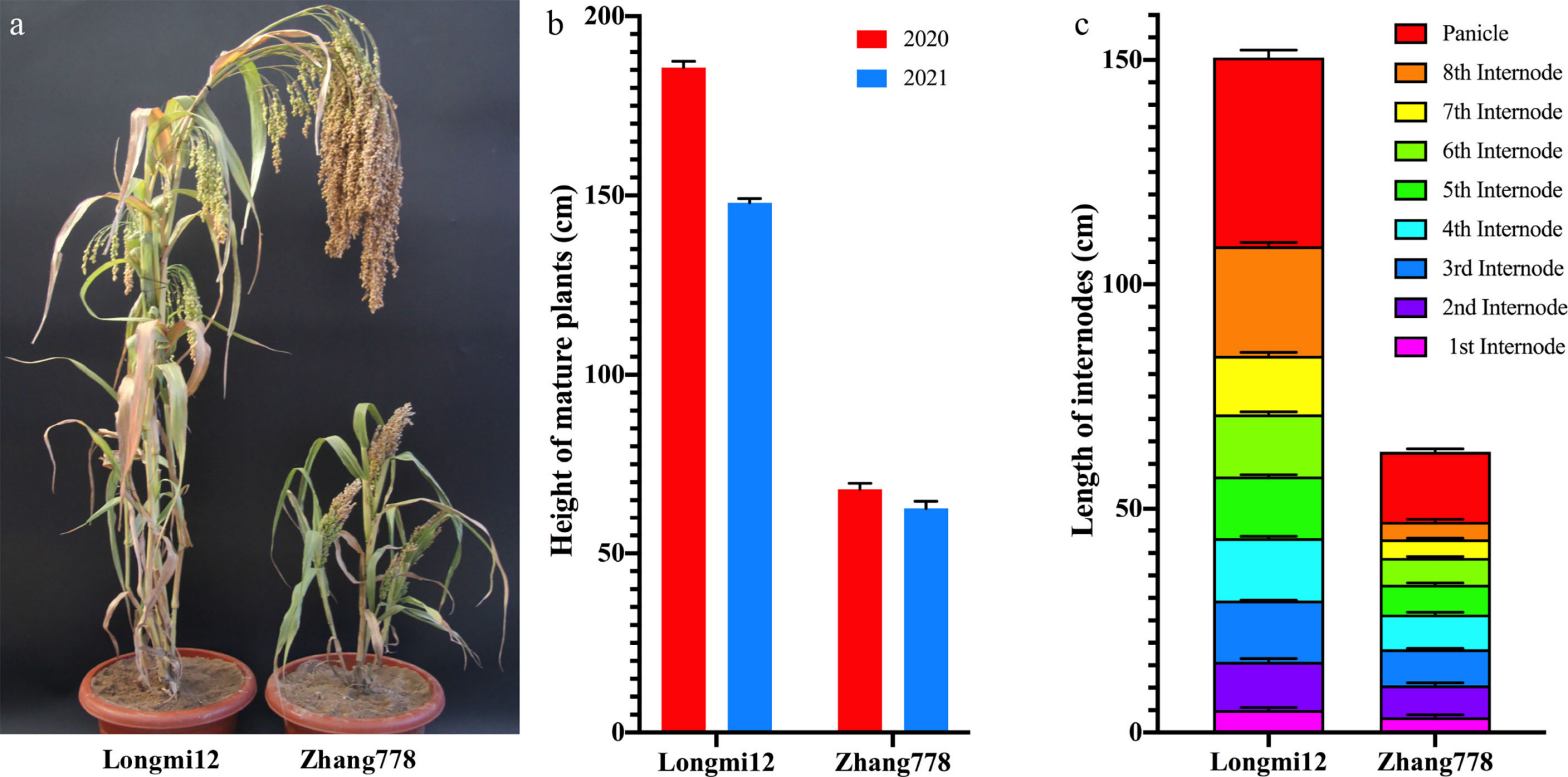
Plant height performance of Longmi12 and Zhang778. **(A)** Mature plants of Longmi12 and Zhang778. **(B)** Comparison of the height of mature Longmi12 and Zhang 778 plants across 2 years. **(C)** Comparison of the length of each internode from Longmi12 and Zhang778.

The plant height of cereal crops is usually determined by the elongation and/or the number of nodes. Here, the dwarf mutant Zhang778 and the normal variety Longmi12, as well as their F_2_ and F_2:3_ progenies, were planted and phenotyped in the same location across 2 years. Compared with the height of Longmi12 that ranged from 163.1 to 188.5 cm, that of mature Zhang778 plants was only about 58.3–72.1 cm (**Figure 1B**). The number of nodes in mature plants was the same in two accessions, suggesting that the dwarf trait in Zhang778 was caused only by the decreased length of the internodes. A comparison of the plant height and the internode length between the two parental lines is shown in **Figure 1**. Besides plant height, the panicle length of Zhang778 was also shorter than that of Longmi12 (**Figures 1A, C**).

The plant heights of the F_2_ and F_2:3_ progenies were continuous and approximately normally distributed and showed no transgressive segregation (**Supplementary Figure S1**). These results suggested that there might be more than one underlying gene and that all favorable alleles were contributed by one parent. To understand the genetic architecture of plant height, we used the major gene plus polygene mixed inheritance model and analyzed the F_2_ population with the R package “SEA V2.0.” The results indicated that 2MG-EA (two major genes with additive effects) was the optimal model, and the heritability of the two major genes was about 88.5% (**Supplementary Table S2**). In addition, the height distribution ranges of the two generations were slightly different (**Supplementary Figure S1**), which was identical to the parental performance, suggesting that environment factors also had an effect on the final height of broomcorn millet.

### Initial mapping of plant height by bulked segregant analysis

**Figure 2:**
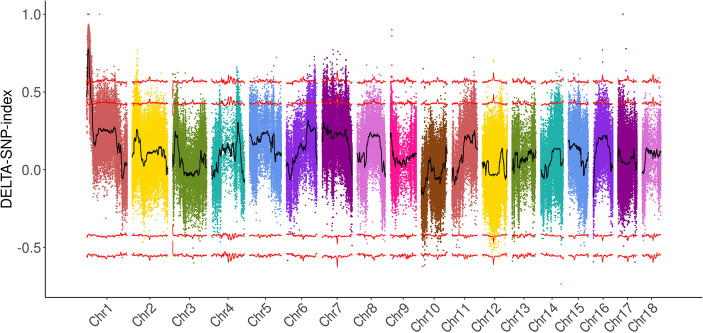
Mapping of a locus controlling plant height in broomcorn millet using the ΔSNP index with the bulked segregant analysis (BSA) strategy.

BSA-seq was carried out to rapidly identify the genomic regions of plant height. A total of 125 M and 348 M paired reads were generated from the two parents and two bulks, respectively. The average sequencing depth of the parents was 22.4×, while that of the bulks was 61.3× (**Supplementary Table S3**). The clean reads were aligned to the reference genome of broomcorn millet (Zou et al., 2019), which revealed that about 97% and 93% of the whole genome in the bulks and parents, respectively, were covered by more than 10× reads. A total of 734,746 SNPs and 84,609 small Indels were identified between the two parents, while 220,363 SNPs and 27,565 small Indels were identified between the two bulks (**Supplementary Table S3**). The ΔSNP index was calculated, and a major region located on Chr1: 1,259,618–6,443,508 was identified with a 99% confidence value (**Figure 2**; **Supplementary Table S4**). The ΔIndel index was also calculated, and an interval on Chr1, which overlapped with the ΔSNP index interval, was identified (**Supplementary Figure S2**; **Table S4**). Overall, the results of BSA-seq revealed a major candidate region of broomcorn millet plant height on Chr1 (hereafter referred to as *PH1.1*), with a physical distance of about 5.18 Mb.

### Fine-Mapping of *PH1.1*

**Figure 3:**
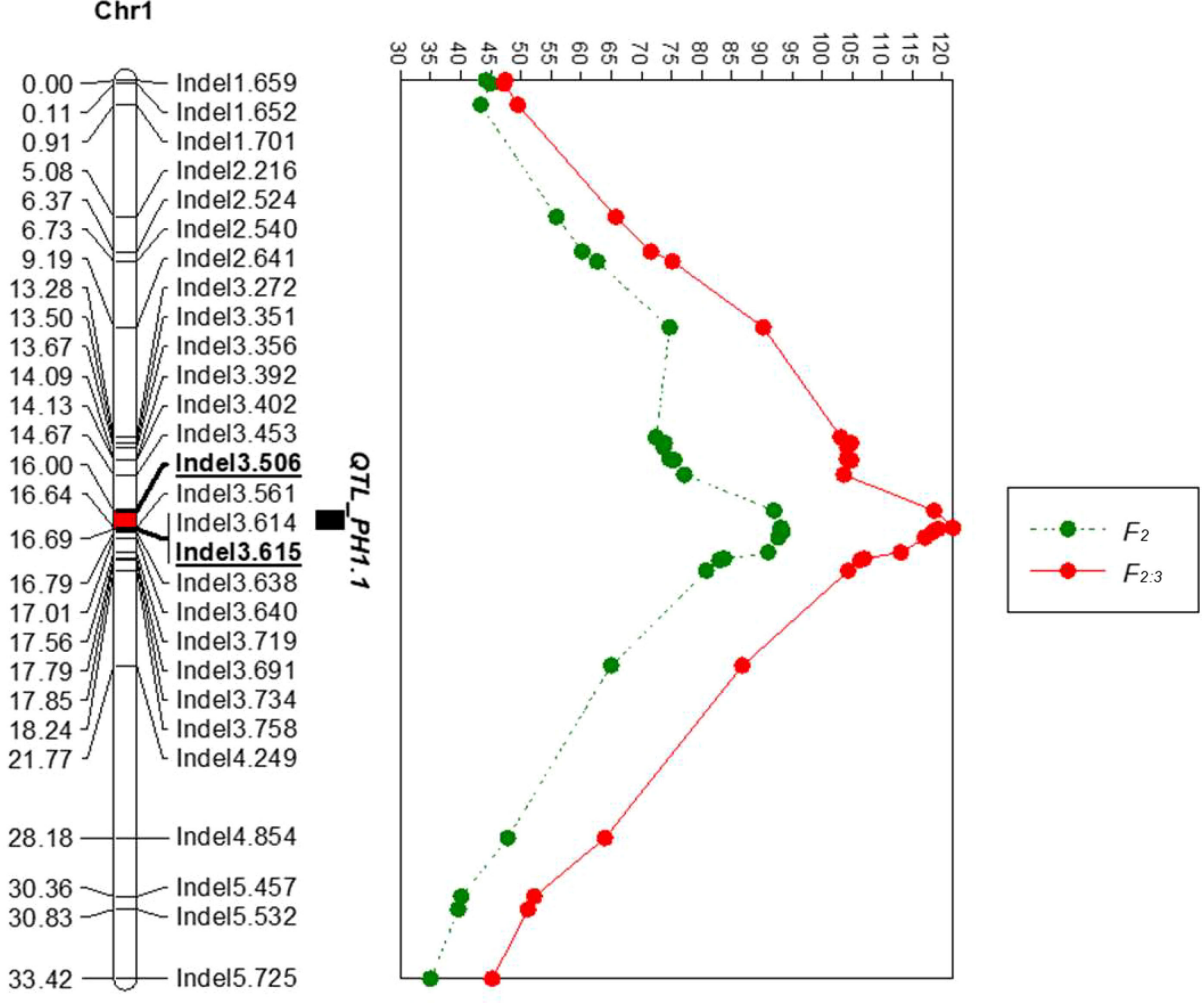
Fine-mapping of *PH1.1* by linkage analysis. The *left panel* shows the linkage map, in which the *red* segment is the fine positioning interval of *PH1.1*. The *right panel* displays the log of odds (LOD) value of each locus in the F_2_ and F_2:3_ generations.

To narrow down the mapping region of *PH1.1*, a total of 68 Indel primers were developed based on the genome resequencing data. The primers were first used to screen for polymorphism between parents. Twenty-eight co-dominant primer pairs with clear bands were then utilized to identify the genotypes of 939 F_2_ individuals (**Supplementary Table S5**) and to construct a high-density genetic map around the region of *PH1.1* (**Figure 3**). The sequences of these primers are listed in **Supplementary Table S6**. QTL analysis was further carried out using the plant height phenotypes from the F_2_ and F_2:3_ populations. The results indicated that the region of *PH1.1* was narrowed down between markers Indel3.506 and Indel3.719 with a phenotypic variance explanation (PVE) value of 36% and an additive effect of 16.68 cm in the F_2_ generation and from marker Indel3.506 to Indel3.614 with a PVE value of 45.2% and an additive effect of 14.59cm in the F_2:3_ generation (**Figure 3**; **Supplementary Table S7**). The corresponding physical distance between Indel3.506 and Indel3.615 was 109 kb (109,478 bp).

### Candidate gene prediction analysis for *PH1.1*

**Figure 4:**
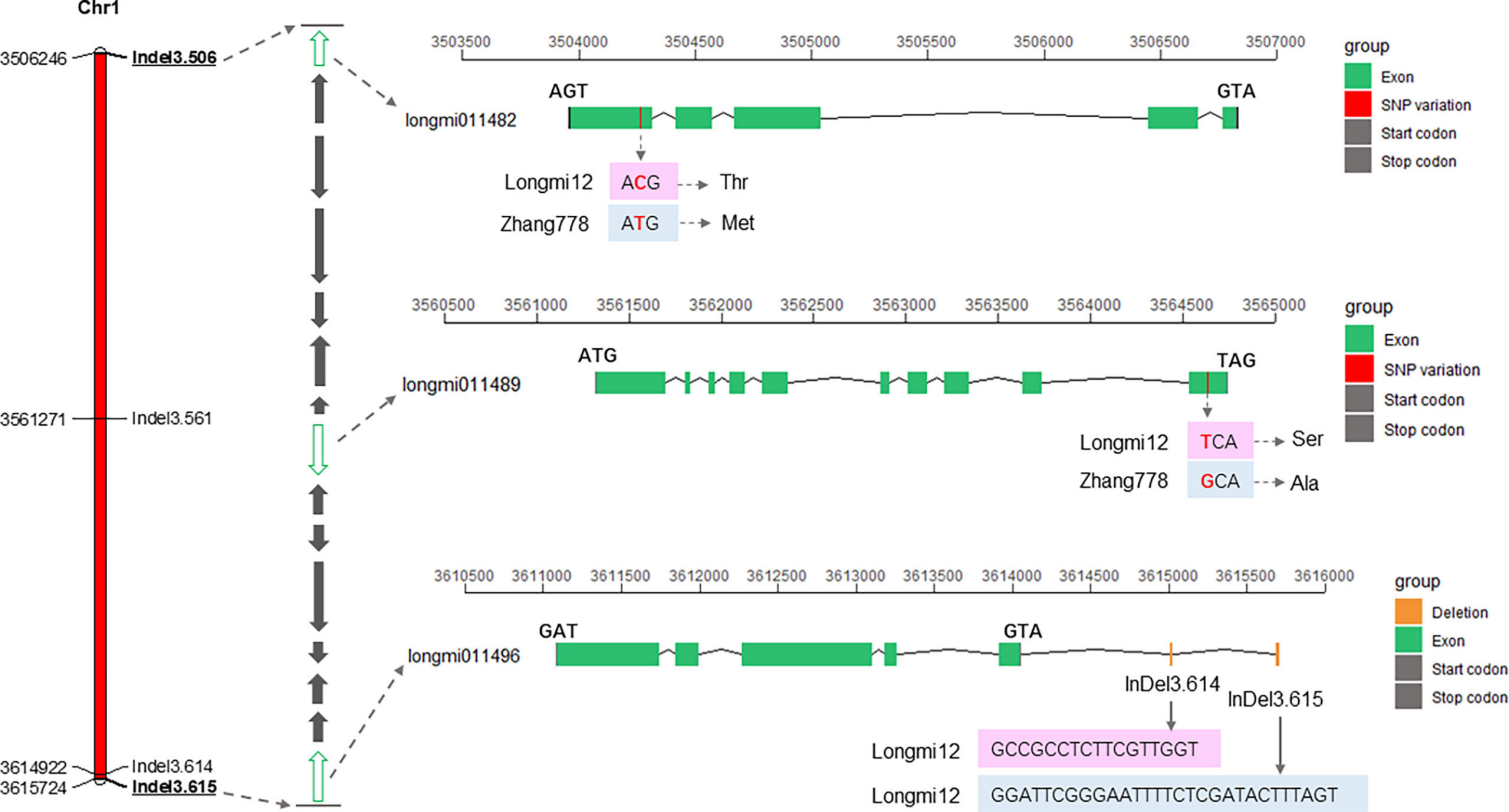
Fine-mapping interval of *PH1.1* and putative candidate genes within the interval. The *left bar* is the physical map of the fine-mapping interval. The *middle arrow* diagram shows the genes in the confidence interval of *PH1.1*, in which *green* indicates the genes *longmi011482*, *longmi011489*, and *longmi011496*, which contained nonsynonymous variation in their coding region and multiple base insertions or deletions in the promoter region. The *right section* displays the variation sites of *longmi011482*, *longmi011489*, and *longmi011496* between Longmi12 and Zhang778.

A total of 15 genes (*longmi011482*–*longmi011496*) were contained in the *PH1.1* interval. Associated genes with ΔSNP or ΔIndel index value equal to 1 in the *PH1.1* interval included *longmi011482*, *longmi011483*, and *longmi011489*, and SNP variants of *longmi011482* and *longmi011489* occurred in the intron and exon, respectively (**Table 1**; **Supplementary Table S8**). Among these genes, 14 were annotated with functions (**Figure 4**; **Table 2**). The sequence alignment of the 15 genes between the parental lines revealed that two nonsynonymous SNPs (Chr1: 3,504,266 and Chr1: 3,564,638) were present in *longmi011482* and *longmi011489* (**Figure 4**). No nonsynonymous variation was detected in the coding regions of the other genes. Furthermore, *longmi011482* encodes a trichome birefringence-like protein, and *longmi011489* encodes the floral homeotic protein *APETALA 2* (*AP2*). Moreover, a few SNPs or Indels were present in the potential promoter regions of *longmi011484*, *longmi011488*, *longmi011489*, and *longmi011496* (**Supplementary Table S8**).

**Table 1:** Identification of sites with ΔSNP/ΔIndel values equal to 1 in the *PH1.1* interval.

Chromosome	Position	Reference	HB allele	SNP/Indel index (HB)	DB allele	SNP/Indel index (DB)	ΔSNP/ΔIndel index	SNP/Indel effect	Gene
Chr1	3,506,727	C	T/T	1	C/C	0	1	Intron	*longmi011482*:(3503959_3506833)
Chr1	3,507,621	T	TA/TA	1	T/T	0	1	Upstream	*longmi011482*:(3503959_3506833)
Chr1	3,507,675	A	G/G	1	A/A	0	1	Upstream	*longmi011482*:(3503959_3506833)
Chr1	3,507,790	T	C/C	1	T/T	0	1	Upstream	*longmi011482*:(3503959_3506833)
Chr1	3,508,129	T	C/C	1	T/T	0	1	Upstream	*longmi011482*:(3503959_3506833)
Chr1	3,511,770	G	A/A	1	G/G	0	1	Downstream	*longmi011483*:(3513538_3517365)
Chr1	3,564,638	T	G/G	1	T/T	0	1	Exon	*longmi011489*:(3561320_3564745)

HB, high bulk; DB, dwarf bulk.

**Table 2:** Annotated function of the genes located in the interval of *PH1.1*.

Gene ID	Pfam domain	Function annotation
*longmi011482*	PMR5 N-terminal domain	Protein trichome birefringence-like 33
*longmi011483*	PMR5 N-terminal domain	Protein trichome birefringence-like 33
*longmi011484*	–	Protein FLX-like 1
*longmi011485*	Histidine phosphatase superfamily (branch 2)	Multiple inositol polyphosphate phosphatase 1
*longmi011486*	NAD-dependent epimerase/dehydratase family; 3-beta hydroxysteroid dehydrogenase/isomerase family; GDP-mannose 4,6-dehydratase	Cinnamoyl-CoA reductase 1
*longmi011487*	Alfin; PHD finger	PHD finger protein ALFIN-LIKE 3
*longmi011488*	Ribosomal protein S12/S23	40S ribosomal protein S23
*longmi011489*	*AP2* domain	Floral homeotic protein *APETALA 2*
*longmi011491*	F-box-like	–
*longmi011492*	Peptidase M1 N-terminal domain	Leukotriene A-4 hydrolase homolog
*longmi011493*	–	Glycine-rich protein A3
*longmi011494*	Heavy metal-associated domain	Protein SODIUM POTASSIUM ROOT DEFECTIVE 2
*longmi011495*	Chalcone–flavonone isomerase	Chalcone–flavonone isomerase
*longmi011496*	Domain of unknown function (DUF4378)	–

### Association test of *PH1.1* among the natural population

**Figure 5:**
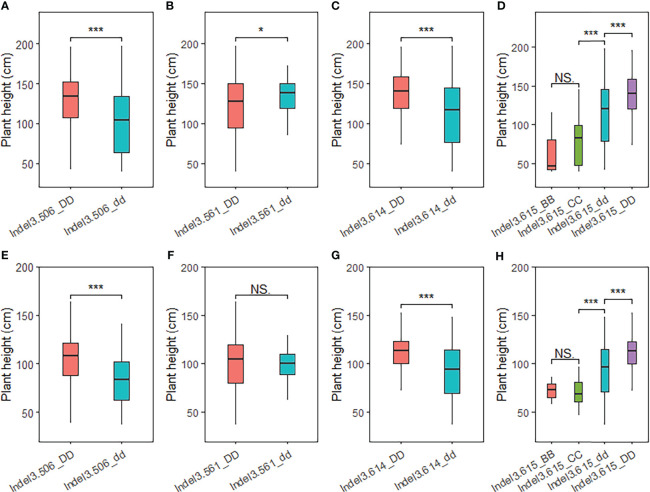
Association test of plant height in the natural population using insertion–deletion (Indel) markers in the *PH1.1* interval. **(A–H)** Association tests of 512 broomcorn millet accessions in 2020 and **(A–D)** and 2021 **(E–H)**. *, *** represent significant differences at P < 0.05 and 0.001, respectively. NS indicates no signifcant difference.

To verify the fine-mapped region of *PH1.1*, a total of 512 broomcorn millet accessions (**Supplementary Table S1**) with various plant heights were genotyped using the four Indel primers in the candidate region (i.e., Indel3.506, Indel3.561, Indel3.614, and Indel3.615). The association test indicated that accessions carrying Longmi12 alleles at both the Indel3.506 and Indel3.614 loci were significantly higher than accessions carrying Zhang778 alleles. In particular, 23 out of the 63 wild germplasm showed genotypes different from those of the two parents at Indel3.615. The plant heights with these genotypes were significantly lower than those of the two parents (**Figure 5**; **Supplementary Table S1**), indicating that Indel3.615 is a key marker that can distinguish different types of broomcorn millet germplasm. Furthermore, both Indel3.614 and Indel3.615 are located upstream of the *longmi011496* gene (**Figure 4**), suggesting that *longmi011496* is also a promising candidate gene. These results confirmed the candidate region of the major QTL *PH1.1* and suggested that *PH1.1* might be a domesticated QTL of broomcorn millet.

## Discussion

### Effective strategy to identify and fine-map major QTLs

With the development of molecular biotechnology in recent years, identifying the QTLs/genes of agronomical traits through forward genetic methods has become a significant part of crop breeding (Song et al., 2007; Ying et al., 2018). Detecting the major QTLs for important traits will not only be helpful to understanding the underlying molecular mechanism but also be favorable to molecular marker-assisted breeding (Liu et al., 2017). Traditional mapping approaches are usually based on a genetic map, the construction of which is time-consuming and labor-intensive (You et al., 2019). High-throughput sequencing technology has enabled not only the assembly of high-quality reference genomes for most crops but also the rapid detection of sequence variations, such as SNPs and Indels. By combining bulk segregation analysis and sequencing approach, BSA-seq has been successfully applied in many crops (Lee et al., 2020; Chen et al., 2021; Wu et al., 2022; Zhang et al., 2022). However, due to the linkage disequilibrium and the incomplete coverage of the sequencing reads, the QTL regions identified using BSA-seq are usually large and need to be narrowed down further (Chen et al., 2021; Zhang et al., 2022). Therefore, the traditional mapping approach cannot be completely replaced by BSA-seq and is still needed in the fine-mapping procedure. Furthermore, the large size of the segregation population is also of significance for further fine-mapping since more recombinants could be obtained from larger populations (Xu et al., 2017). In this study, we firstly detected the *PH1.1* region using BSA-seq, then constructed a genetic map to fine-map *PH1.1* using nearly a thousand progenies, and *PH1.1* was finally successfully narrowed into a 0.109-Mb region. The strategy used in the present study accelerated the identification of major QTLs and could be applied in the detection of other QTLs in broomcorn millet.

### *PH1.1* is a major domesticated QTL

The domestication of broomcorn millet from wild species to modern cultivated varieties has greatly increased the size of the panicle and boosted production. The continuous artificial selection of high yield resulted in the tall plant architecture of cultivated broomcorn millet varieties since plant height is usually positively correlated with panicle size in cereal plants (Zhang et al., 2006; Liu et al., 2013; Chen et al., 2014). Here, a major QTL, *PH1.1*, was identified across two environments and explained about 45% of the variation in the plant height of broomcorn millet. This result indicated that *PH1.1* is a stable locus with a large contribution. Through genotyping with the three Indel markers in the *PH1.1* region, broomcorn millet accessions with different alleles were significantly segregated according to plant height. In particular, Indel3.615, an insertion/deletion marker in the promoter region of the *longmi011496* gene, can clearly distinguish wild broomcorn millet germplasm from the two parental lines on plant height. This association test suggested that the dwarf allele of *PH1.1* existed in the wild broomcorn millet accessions, although the dwarf parent Zhang778 was obtained from EMS mutation.

The prediction of the inheritance model has been proven to be powerful in understanding the genetic architecture of quantitative traits (Wang et al., 2001). In the current study, the continuous distribution of plant height in the F_2_ population indicates multiple underlying genes. Two major genes were predicted to control plant height in the F_2_ population, with a heritability of about 88.5%. Therefore, besides *PH1.1*, another major gene and a few minor genes possibly regulated the plant height of the dwarf mutant Zhang778, which needed further exploration.

### Possible candidate gene for *PH1.1*

In the confidence interval of *PH1.1*, five of the 15 genes showed sequence variation in the coding region or the potential promoter region. Among them, *longmi011489* encodes an *AP2* transcriptional factor harboring variations in both the coding and promoter regions. A few mutants of the *AP2* genes have been reported as candidate genes of dwarf plants, such as *ddf1* in *Arabidopsis* (Magome et al., 2004), *ndl1* in rice (Kusnandar et al., 2022), and *dil1* in maize (Jiang et al., 2012). *AP2* genes have been reported to play a significant role in GA signaling pathways, which are involved in the growth and development of plants (Magome et al., 2004; Xue et al., 2022). In a previous study, Zhang778 was proven to be caused by a mutation of the genes related to GA synthesis (Zhang et al., 2020), which is consistent with the function of *AP2* genes. In addition, another previous study detected 21 genes involved in the domestication of broomcorn millet, 19 of which were annotated into the *AP2* gene family (Li et al., 2021). As mentioned above, *PH1.1* was probably a domesticated QTL, consistent with the roles of the *AP2* gene family involved in the domestication of broomcorn millet. Another candidate gene, *longmi011482*, encodes a trichome birefringence-like protein related to cell wall polysaccharide *O*-acetylation in *Arabidopsis* (Schultink et al., 2015) and fiber development in upland cotton (Yu and Gervers, 2019). Thus, it may participate in the development of the cell wall to affect the elongation of the broomcorn millet internode and then regulate plant height. Furthermore, *longmi011496* is an unknown function gene; however, in the association analysis carried out in this study, the base insertion or deletion in its promoter region was closely related to its plant height and can significantly distinguish wild accessions based on the height of the dwarf plant, indicating that the gene may be a specific gene regulating plant height in broomcorn millet. Overall, their functions still need further verification.

### Diagnostic markers of *PH1.1* could be applied in MAS

Diagnostic markers are molecular markers that are significantly linked with one or more traits, which help in the early and rapid identification of a target phenotype (Chakdar et al., 2019). Diagnostic markers have been successfully employed in many traits of various plants, such as the rust resistance trait in wheat (Liu et al., 2021) and common wheat (Wu et al., 2018), the testa color in cultivated peanut (Chen et al., 2021), and the bacterial wilt trait in tomato (Abebe et al., 2020). In the present study, three Indel markers located in the *PH1.1* region were validated to be closely linked with the plant height of broomcorn millet using a total of 512 accessions. As do other cereal crops, high broomcorn millet varieties usually suffer from lodging; hence, breeding new broomcorn millet varieties with optimal height will be of great importance. With the three diagnostic markers, particularly Indel3.615, the plant height of new broomcorn varieties could be distinguished at the seedling stages. Therefore, the diagnostic markers in the present study laid a foundation for the molecular breeding of broomcorn millet and could be directly applied in its MAS breeding.

## Data availability statement

The datasets presented in this study can be found in online repositories. The names of the repository/repositories and accession number(s) can be found in the article/**Supplementary Material**.

## Author contributions

TL and XL performed data analysis and drafted the manuscript. ZZ and TY designed the experiment and revised the manuscript. TL and JH constructed the F_2_ and F_2:3_ populations. KD, LZ, and RR collected the phenotype. WP completed the collection of population genotypes. All authors contributed to the article and approved the submitted version.

## Funding

This study was financially supported by the China Agriculture Research System (CARS-06-14.5-A8); Special Project of Agricultural Science and Technology innovation of GAAS (2021GAAS02); and the Top-Notch Talent Project in Gansu Province (2021).

## Acknowledgments

The authors would like to thank Prof. Ping Lu and Dr. Minxuan Liu (Chinese Academy of Agricultural Sciences) for providing seeds and basic information on the 512 broomcorn millet accessions for this study.

## Conflict of interest

The authors declare that the research was conducted in the absence of any commercial or financial relationships that could be construed as a potential conflict of interest.

## Publisher’s note

All claims expressed in this article are solely those of the authors and do not necessarily represent those of their affiliated organizations, or those of the publisher, the editors and the reviewers. Any product that may be evaluated in this article, or claim that may be made by its manufacturer, is not guaranteed or endorsed by the publisher.

## References

[B1] Abebe A. M. Choi J. Kim Y. Oh C. S. Yeam I. Nou I. S.. (2020). Development of diagnostic molecular markers for marker-assisted breeding against bacterial wilt in tomato. Breed Sci. 70, 462–473. doi: 10.1270/jsbbs.20027 32968349 PMC7495205

[B2] Chakdar H. Goswami S. K. Singh E. Choudhary P. Yadav J. Kashyap P. L.. (2019). noxB-based marker for alternaria spp.: A new diagnostic marker for specific and early detection in crop plants. Biotech 9, 249. doi: 10.1007/s13205-019-1779-4 PMC6548793 31218173

[B3] Chen H. Chen X. Xu R. Liu W. Liu N. Huang L.. (2021). Fine-mapping and gene candidate analysis for AhRt1, a major dominant locus responsible for testa color in cultivated peanut. Theor. Appl. Genet. 134, 3721–3730. doi: 10.1007/s00122-021-03924-w 34379146

[B4] Chen S. Gao R. Wang H. Wen M. Xiao J. Bian N.. (2014). Characterization of a novel reduced height gene (Rht23) regulating panicle morphology and plant architecture in bread wheat. Euphytica 203, 583–594. doi: 10.1007/s10681-014-1275-1

[B5] Das S. Khound R. Santra M. Santra D. K. (2019). Beyond bird feed: Proso millet for human health and environment. Agricult. MDPI 9 (3), 1–19. doi: 10.3390/agriculture9030064

[B6] Hunt H. V. Badakshi F. Romanova O. Howe C. J. Jomes M. K. Heslop-Harrison J. S. P. (2014). Reticulate evolution in *Panicum* (Poaceae): The origin of tetraploid broomcorn millet, p. miliaceum. J. Exp. Bot. 65, 3165–3175. doi: 10.1093/jxb/eru161 24723408 PMC4071833

[B7] Itoh H. Tatsumi T. Sakamoto T. Otomo K. Toyomasu T. Kitano H.. (2004). A rice semi-dwarf gene, tan-ginbozu (D35), encodes the gibberellin biosynthesis enzyme, ent-kaurene oxidase. Plant Mol. Biol. 54, 533–547. doi: 10.1023/B:PLAN.0000038261.21060.47 15316288

[B8] Jiang F. Guo M. Yang F. Duncan K. Jackson D. Rafalski A.. (2012). Mutations in an AP2 transcription factor-like gene affect internode length and leaf shape in maize. PloS One 7, e37040. doi: 10.1371/journal.pone.0037040 22649507 PMC3359370

[B9] Koppolu R. Jiang G. Milner S. G. Muqaddasi Q. H. Rutten T. Himmelbach A.. (2022). The barley mutant multiflorus2.b reveals quantitative genetic variation for new spikelet architecture. Theor. Appl. Genet. 135, 571–590. doi: 10.1007/s00122-021-03986-w 34773464 PMC8866347

[B10] Kusnandar A. S. Itoh J. I. Sato Y. Honda E. Hibara K. I. Kyozuka J.. (2022). Narrow and dwarf leaf 1, the ortholog of arabidopsis enhancer of shoot Regeneration1/Dornroschen, mediates leaf development and maintenance of the shoot apical meristem in oryza sativa l. Plant Cell Physiol. 63, 265–278. doi: 10.1093/pcp/pcab169 34865135

[B11] Lee S. B. Kim J. E. Kim H. T. Lee G. M. Kim B. S. Lee J. M. (2020). Genetic mapping of the c1 locus by GBS-based BSA-seq revealed pseudo-response regulator 2 as a candidate gene controlling pepper fruit color. Theor. Appl. Genet. 133, 1897–1910. doi: 10.1007/s00122-020-03565-5 32088729

[B13] Li H. Durbin R. (2009). Fast and accurate short read alignment with burrows-wheeler transform. Bioinformatics 25, 1754–1760. doi: 10.1093/bioinformatics/btp324 19451168 PMC2705234

[B12] Li C. Liu M. Sun F. Zhao X. He M. Li T.. (2021). Genetic divergence and population structure in weedy and cultivated broomcorn millets (Panicum miliaceum l.) revealed by specific-locus amplified fragment sequencing (SLAF-seq). Front. Plant Sci. 12, 688444. doi: 10.3389/fpls.2021.688444 34249058 PMC8264369

[B16] Liu Y. Chen H. Li C. X. Zhang L. R. Shao M. Q. Pang Y. H.. (2021). Development of diagnostic markers for a wheat leaf rust resistance gene Lr42 using RNA-sequencing. Crop J. 9, 1357–1366. doi: 10.1016/j.cj.2021.02.012

[B14] Liu T. Liu H. Zhang H. Xing Y. (2013). Validation and characterization of Ghd7.1, a major quantitative trait locus with pleiotropic effects on spikelets per panicle, plant height, and heading date in rice (Oryza sativa l.). J. Integr. Plant Biol. 55, 917–927. doi: 10.1111/jipb.12070 23692054

[B15] Liu X. Teng Z. Wang J. Wu T. Zhang Z. Deng X.. (2017). Enriching an intraspecific genetic map and identifying QTL for fiber quality and yield component traits across multiple environments in upland cotton (*Gossypium hirsutum* l.). Mol. Genet. Genomics 292, 1281–1306. doi: 10.1007/s00438-017-1347-8 28733817

[B17] Lu H. Zhang J. Liu K. B. Wu N. Li Y. Zhou K.. (2009). Earliest domestication of common millet (*Panicum miliaceum*) in East Asia extended to 10,000 years ago. Proc. Natl. Acad. Sci. U.S.A. 106, 7367–7372. doi: 10.1073/pnas.0900158106 19383791 PMC2678631

[B18] Magome H. Yamaguchi S. Hanada A. Kamiya Y. Oda K. (2004). Dwarf and delayed-flowering 1, a novel arabidopsis mutant deficient in gibberellin biosynthesis because of overexpression of a putative AP2 transcription factor. Plant J. 37, 720–729. doi: 10.1111/j.1365-313X.2003.01998.x 14871311

[B19] Makarevitch I. Thompson A. Muehlbauer G. J. Springer N. M. (2012). Brd1 gene in maize encodes a brassinosteroid c-6 oxidase. PloS One 7, e30798. doi: 10.1371/journal.pone.0030798 22292043 PMC3266906

[B20] Sasaki A. Ashikari M. Ueguchi-Tanaka M. Itoh H. Nishimura A. Swapan D.. (2002). Green revolution: a mutant gibberellin-synthesis gene in rice. Nature 416, 701–702. doi: 10.1038/416701a 11961544

[B21] Sazuka T. Kamiya N. Nishimura T. Ohmae K. Sato Y. Imamura K.. (2009). A rice tryptophan deficient dwarf mutant, tdd1, contains a reduced level of indole acetic acid and develops abnormal flowers and organless embryos. Plant J. 60, 227–241. doi: 10.1111/j.1365-313X.2009.03952.x 19682283

[B22] Schultink A. Naylor D. Dama M. Pauly M. (2015). The role of the plant-specific altered xyloglucan9 protein in arabidopsis cell wall polysaccharide o-acetylation. Plant Physiol. 167 (4), 1271–u243. doi: 10.1104/pp.114.256479 25681330 PMC4378174

[B23] Shi J. Ma X. Zhang J. Zhou Y. Liu M. Huang L.. (2019). Chromosome conformation capture resolved near complete genome assembly of broomcorn millet. Nat. Commun. 10, 464. doi: 10.1038/s41467-018-07876-6 30683940 PMC6347627

[B24] Song X. Huang W. Shi M. Zhu M. Lin H. (2007). A QTL for rice grain width and weight encodes a previously unknown RING-type E3 ubiquitin ligase. Nat. Genet. 39, 623–630. doi: 10.1038/ng2014 17417637

[B25] Van Ooijen J. W. (2006). JoinMap 4.0: software for the calculation of genetic linkage maps in experimental populations (Wageningen: Kyazma BV).

[B26] Van Ooijen J. (2009). MapQTL 6, software for the mapping of quantitative trait loci in experimental populations of diploid species (Wageningen: Kyazma BV).

[B27] Voorrips R. (2002). MapChart: software for the graphical presentationof linkage maps and QTLs. J. Hered. 93, 77–78. doi: 10.1093/jhered/93.1.77 12011185

[B28] Wang J. Podlich D. W. Cooper M. DeLacy I. H. (2001). Power of the joint segregation analysis method for testing mixed major-gene and polygene inheritance models of quantitative traits. Theor. Appl. Genet. 103, 804–816. doi: 10.1007/s001220100628

[B29] Wu J. Huang S. Zeng Q. Liu S. Wang Q. Mu J.. (2018). Comparative genome-wide mapping versus extreme pool-genotyping and development of diagnostic SNP markers linked to QTL for adult plant resistance to stripe rust in common wheat. Theor. Appl. Genet. 131, 1777–1792. doi: 10.1007/s00122-018-3113-7 29909527

[B30] Wu L. Wang H. Liu S. Liu M. Liu J. Wang Y.. (2022). Mapping of CaPP2C35 involved in the formation of light-green immature pepper (Capsicum annuum l.) fruits *via* GWAS and BSA. Theor. Appl. Genet. 135, 591–604. doi: 10.1007/s00122-021-03987-9 34762177

[B32] Xue Y. Zhang Y. Shan J. Ji Y. Zhang X. Li W.. (2022). Growth repressor GmRAV binds to the GmGA3ox promoter to negatively regulate plant height development in soybean. Int. J. Mol. Sci. 23, 1721. doi: 10.3390/ijms23031721 35163641 PMC8836252

[B31] Xu P. Gao J. Cao Z. Chee P. W. Guo Q. Xu Z.. (2017). Fine mapping and candidate gene analysis of qFL-chr1, a fiber length QTL in cotton. Theor. Appl. Genet. 130, 1309–1319. doi: 10.1007/s00122-017-2890-8 28361363

[B33] Yang X. Wan Z. Perry L. Lu H. Wang Q. Zhao C.. (2012). Early millet use in northern China. Proc. Natl. Acad. Sci. U.S.A. 109, 3726–3730. doi: 10.1073/pnas.1115430109 22355109 PMC3309722

[B34] Ying J.-Z. Ma M. Bai C. Huang X.-H. Liu J.-L. Fan Y.-Y.. (2018). TGW3 , a major QTL that negatively modulates grain length and weight in rice. Mol. Plant 11, 750–753. doi: 10.1016/j.molp.2018.03.007 29567450

[B35] You Q. Yang X. P. Peng Z. Islam M. S. Sood S. Luo Z. L.. (2019). Development of an axiom Sugarcane100K SNP array for genetic map construction and QTL identification. Theor. Appl. Genet. 132, 2829–2845. doi: 10.1007/s00122-019-03391-4 31321474

[B36] Yu J. Z. Gervers K. A. (2019). Genomic analysis of marker-associated fiber development genes in upland cotton (*Gossypium hirsutum* l). Euphytica 215 (4), 74. doi: 10.1007/s10681-019-2388-3

[B37] Zhang B. Liu X. Guo Y. Jia X. Zhao Y. Dai L.. (2020). Investigation on agronomic characters of dwarf mutant 778 in broomcorn millet (*Panicum miliaceum* l.) and analysis of its sensitivity to GA. Agric. Biotechnol. 9, 7–11. doi: 10.19759/j.cnki.2164-4993.2020.04.003

[B39] Zhang Y. Luo L. Xu C. Zhang Q. Xing Y. (2006). Quantitative trait loci for panicle size, heading date and plant height co-segregating in trait-performance derived near-isogenic lines of rice (Oryza sativa). Theor. Appl. Genet. 113, 361–368. doi: 10.1007/s00122-006-0305-3 16791702

[B40] Zhang Z.-S. Xiao Y.-H. Luo M. Li X.-B. Luo X.-Y. Hou L.. (2005). Construction of a genetic linkage map and QTL analysis of fiber-related traits in upland cotton (Gossypium hirsutum l.). Euphytica 144, 91–99. doi: 10.1007/s10681-005-4629-x

[B38] Zhang K. Yuan M. Xia H. He L. Ma J. Wang M.. (2022). BSAseq and genetic mapping reveals AhRt2 as a candidate gene responsible for red testa of peanut. Theor. Appl. Genet. 135, 1529–1540. doi: 10.1007/s00122-022-04051-w 35166897

[B41] Zou C. Li L. Miki D. Li D. Tang Q. Xiao L.. (2019). The genome of broomcorn millet. Nat. Commun. 10, 436. doi: 10.1038/s41467-019-08409-5 30683860 PMC6347628

