# Supplementary figures and images for: Identification and fine-mapping of a major QTL (PH1.1) conferring plant height in broomcorn millet (Panicum miliaceum)
Source: Front Plant Sci. 2022 Oct 11;13:1010057. doi: 10.3389/fpls.2022.1010057 (PMC9593001; doi:10.3389/fpls.2022.1010057)

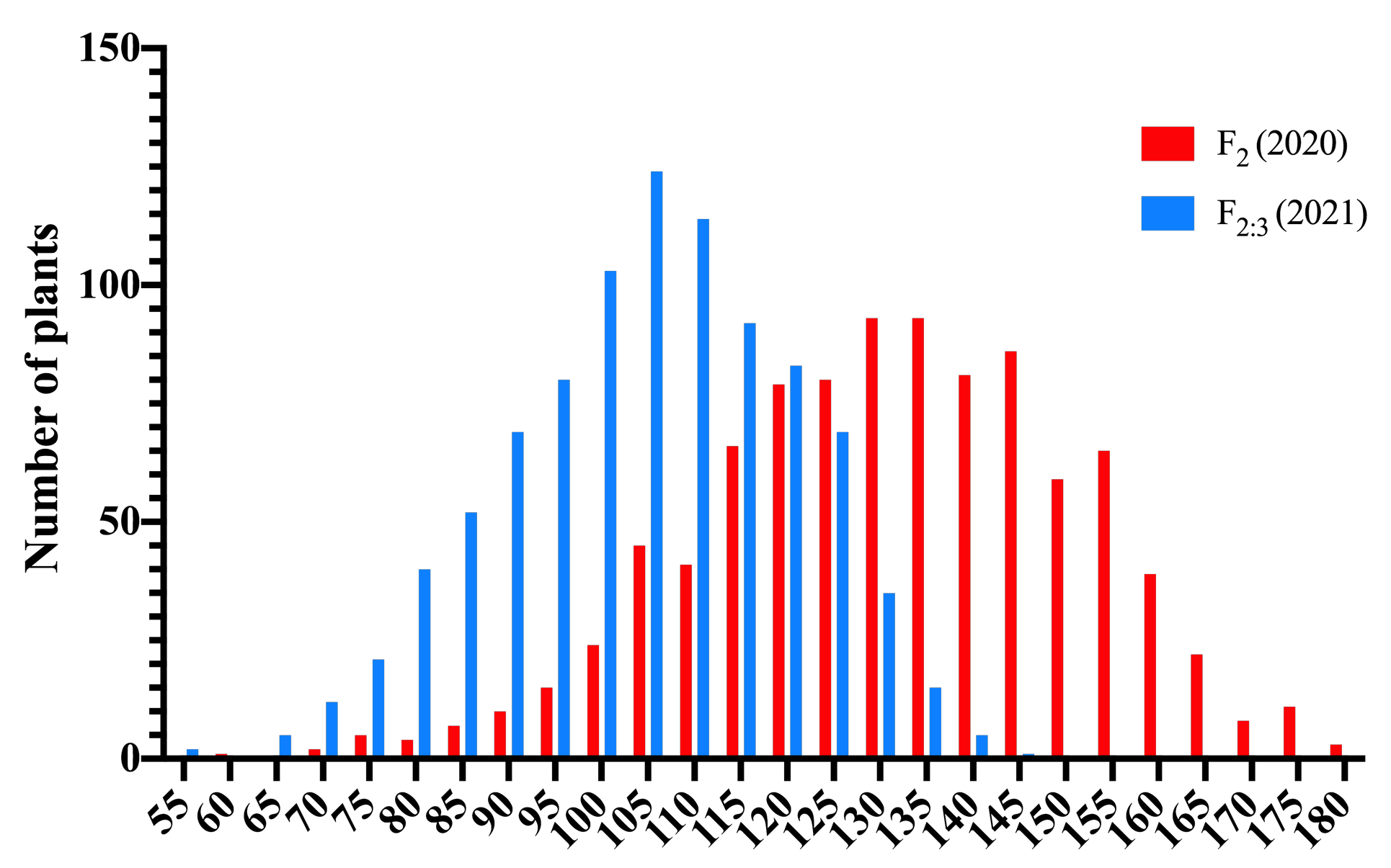

Supplement: Supplementary file 1 [file Image_1.jpeg]

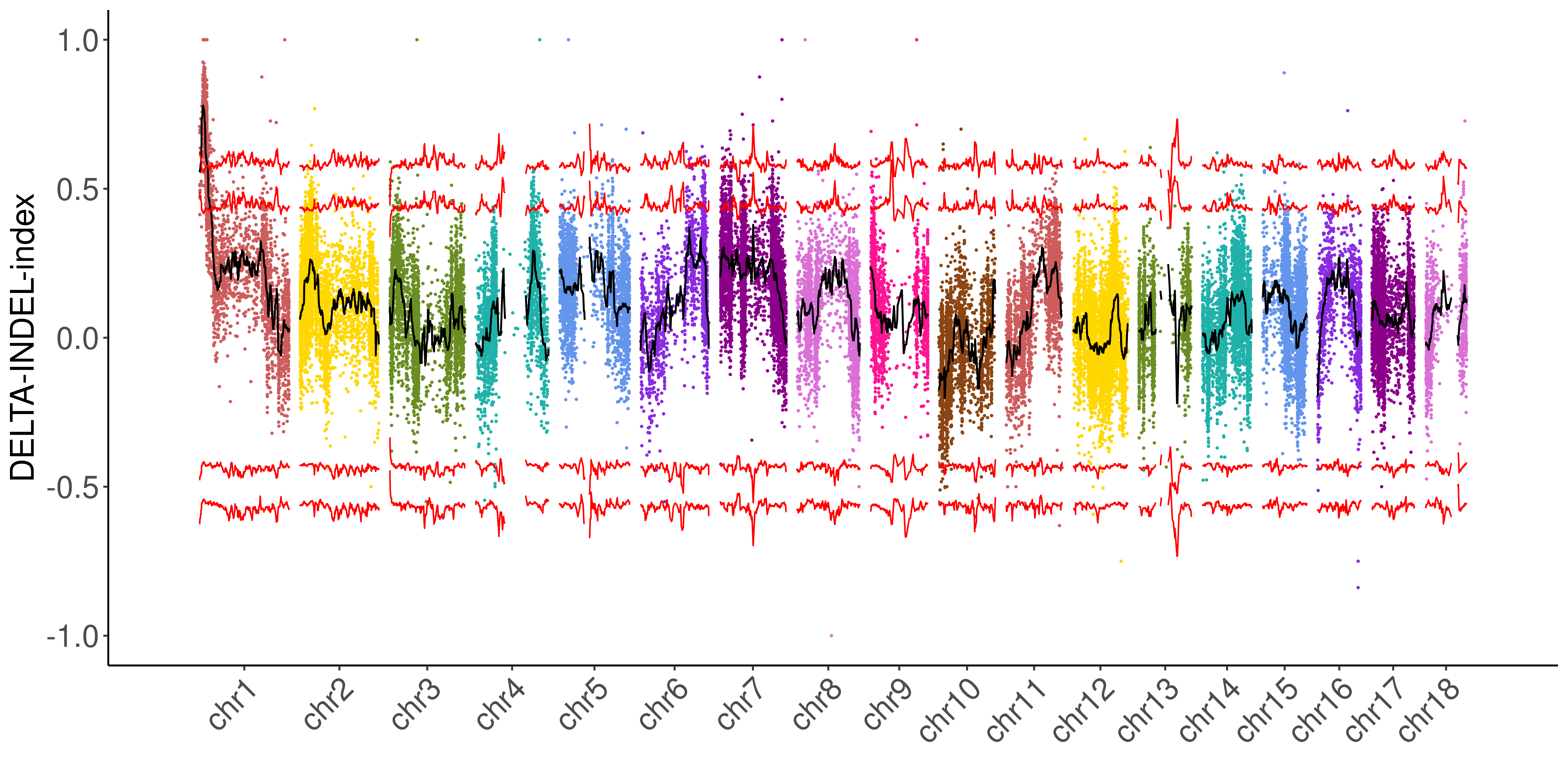

Supplement: Supplementary file 2 [file Image_2.jpeg]
